# Determinants of the renewable energy consumption: The case of Asian countries

**DOI:** 10.1016/j.heliyon.2023.e22696

**Published:** 2023-11-24

**Authors:** Pham Xuan Hoa, Vu Ngoc Xuan, Nguyen Thi Phuong Thu

**Affiliations:** a Department of Public Finance, School of Banking and Finance, National Economics University, Viet Nam; b Department of Microeconomics, Faculty of Economics, National Economics University, Viet Nam; c Department of Public Economics, Faculty of Development Economics, National Economics University, Viet Nam

**Keywords:** electricity consumption, Fossil fuel consumption, Foreign direct investment inflows, Economic growth

## Abstract

In recent year, Asia has rapid economic growth and policy makers care about the renewable energy consumption. The motivation about the choice of variables with theory and empirical backing on the subject in the manuscript is the theory of Cobb- Douglas function and determinants of renewable energy consumption in the Asian countries. This paper investigates the nexus of electricity consumption, fossil fuel consumption, foreign direct investment inflows, economic growth, and renewable energy consumption in Asian countries. Utilizing panel data analysis, the study finds significant interrelationships among these factors and highlights their implications for energy policy and sustainable development in the region. This article aims to use the Cobb- Douglas Model to analyze the factors affecting renewable energy consumption at Asian developed countries for sustainable development goals. This study collected data from World Bank in the period 2000–2020 of 06 developed countries such as Japan, Korea, Singapore, Hong Kong, Israel and China. The empirical research results show that the electricity consumption, fossil fuel consumption, foreign direct investment inflows, economic growth affect the environmental pollution in 06 Asian developed countries. The electricity consumption, foreign direct investment inflows, economic growth positively affect to the renewable energy consumption. If the electricity consumption increases 1 % then the renewable energy consumption up 0.6 %; if the foreign direct investment inflows up 1 % then the renewable energy consumption up 0.82 %; If the economic growth up 1 % then renewable energy up 2.73 %. In addition, fossil fuel consumption negatively affects the renewable energy consumption. If the fossil fuel consumption increases 1 % then the renewable energy consumption down 0.26 %. The paper offers conclusions and recommendations for Asian countries to address these interconnected issues and transition towards a more sustainable energy future.

## Highlights

• This paper investigates the nexus of electricity consumption, fossil fuel consumption, foreign direct investment (FDI) inflows, economic growth, and renewable energy consumption in Asian countries. Utilizing panel data analysis, the study finds significant interrelationships among these factors and highlights their implications for energy policy and sustainable development in the region.

## 1 Introduction

In recent decades, Asia has experienced rapid economic growth, driven in part by the increasing consumption of energy resources, particularly electricity and fossil fuels. This growth has led to significant environmental challenges, such as air pollution and climate change, which have prompted countries in the region to explore cleaner and more sustainable energy sources, including renewable energy. Additionally, foreign direct investment (FDI) inflows have played a crucial role in driving economic growth and facilitating the adoption of new energy technologies in the region. The purpose of the paper is to specify why understanding the determinants of renewable energy is important. The objective of the paper is to provide background information on the current state of renewable energy, emphasizing the global or local context, especially in Asian countries.

Given these trends, understanding the nexus of electricity consumption, fossil fuel consumption, FDI inflows, economic growth, and renewable energy consumption in Asia is crucial for designing effective policies and strategies to address the region's energy challenges and ensure sustainable development. This paper contributes to this understanding by examining the interrelationships among these factors and their implications for energy policy and sustainable development in Asian countries. The world faces the warming of the atmosphere due to the environmental pollution. The scientists and policy makers care about the down the environment pollution for the sustainable development goals.

Aghasafari et al. studied CO_2_ Emissions, Export and Foreign Direct Investment: Empirical Evidence from Middle East and North Africa Region [1]. Al Afif et al. studied Feasibility and Optimal Sizing Analysis of Hybrid Renewable Energy Systems: A Case Study of Al-Karak, Jordan [2]. There are a gap research about using the Cobb-Douglas model to regression the factors effecting the environmental pollution at Asian developed countries and how the renewable energy consumption, FDI inflows, economic growth, import of goods and services, export of goods and services, population can effect to CO_2_ emission. Therefore, this research focuses on the relationship between electricity consumption, fossil fuel consumption, FDI inflows, economic growth and renewable energy consumption in 06 Asian developed countries. These countries are Japan, Korea, Singapore, Hong Kong, Israel and China.

Electricity energy consumption refers to the amount of electrical energy used by households, industries, and other sectors of the economy. It is typically measured in units of kilowatt-hours (kWh) or megawatt-hours (MWh) [1].

Fossil fuel energy consumption refers to the use of non-renewable resources such as coal, oil, and natural gas to generate energy. This type of energy consumption contributes significantly to greenhouse gas emissions, which have been linked to climate change [2].

FDI inflows refer to foreign direct investment, which is the investment made by foreign companies or individuals into a country's economy. This type of investment can help to spur economic growth and development by providing funding for new businesses, infrastructure projects, and other initiatives [3].

Import of goods and services refers to the purchase of goods and services from other countries, while export of goods and services refers to the sale of goods and services to other countries. These metrics can be used to track a country's trade balance and economic competitiveness [4].

Economic growth refers to the up in a country's Gross Domestic Product (GDP) over time. This growth can be driven by factors such as investment, innovation, and productivity improvements [5].

Renewable energy consumption refers to the use of sources of energy that are replenished naturally, such as solar, wind, hydro, and geothermal energy. This type of energy consumption is considered more sustainable than fossil fuel energy consumption, as it does not deplete finite resources or contribute to greenhouse gas emissions [6].

There are various relationships between the concepts you have mentioned, and some of the key ones are:

Electricity energy consumption is often closely linked to economic growth, as more energy is needed to power factories, homes, and other sectors of the economy. However, if the majority of electricity is generated from fossil fuels, then this can also lead to higher CO_2_ emissions, which can have negative impacts on the environment and public health.

Fossil fuel energy consumption is often associated with higher CO_2_ emissions, which contribute to climate change. In contrast, renewable energy consumption can help to reduce greenhouse gas emissions and mitigate the impacts of climate change.

FDI inflows can help to spur economic growth and development by providing funding for new businesses and infrastructure projects. However, if the majority of FDI is directed towards industries that rely heavily on fossil fuels, then this can lead to higher greenhouse gas emissions.

Overall, the relationships between these concepts can be complex and interconnected, and require a holistic approach to understanding and managing them effectively. Countries that prioritize sustainable development and invest in renewable energy, energy efficiency, and low-carbon technologies are likely to see long-term economic and environmental benefits. Therefore, this paper use the Stata 17.0 to estimate the factors effecting the renewable energy consumption in 06 Asian developed countries such as Japan, Korea, Singapore, Hong Kong, Israel and China.

In addressing these research gaps, scholars can contribute to a more comprehensive and nuanced understanding of renewable energy consumption, paving the way for effective policies and strategies to accelerate the transition to a sustainable energy future.

The study contain 05 section: introduction-section 01, literature review-section 02, data and research methodology-section 03, the research results-section 04 and the final one-conclusion – section 06.

## 2 Literature review

### 2.1 Related studies

A substantial body of literature has investigated the relationship between electricity consumption and economic growth, with mixed findings. Some studies have found a positive relationship between electricity consumption and economic growth, suggesting that increased electricity consumption is necessary for economic development. Other studies have found a negative or insignificant relationship, indicating that electricity consumption may not be the primary driver of economic growth [2].

The relationship between fossil fuel consumption and economic growth has also been extensively studied, with most studies finding a positive relationship between the two variables [7]. However, some studies have found a negative relationship, suggesting that high levels of fossil fuel consumption may hinder economic growth due to negative externalities such as pollution and resource depletion [6].

The literature on the relationship between FDI inflows, economic growth, and renewable energy consumption has revealed several important relationships and trends. FDI inflows have been found to positively influence economic growth in developing countries, primarily through technology transfer, knowledge spillovers, and productivity improvements [3]. Moreover, FDI can stimulate innovation in host countries by fostering research and development (R&D) activities, enhancing human capital, and promoting competition [8].

Numerous investigations within the extant literature have scrutinized the nexus between energy utilization and economic growth. Nevertheless, there exists a paucity of empirical studies that have systematically examined the association between the utilization of renewable energy and the tangible Gross Domestic Product (GDP). The divergence in outcomes among these studies emanates from variances in research methodologies and temporal frameworks. Conclusions drawn from extant investigations reveal three predominant causal relationships: unidirectional causation from renewable energy use to GDP or vice versa, indicating bidirectional causality, and the absence of a causal relationship regarding growth [8].

Empirical inquiry into the correlation between energy utilization and economic growth reveals four hypotheses: firstly, the growth hypothesis, constituting 29 % of all empirical studies, posits a unidirectional causation from energy consumption to economic growth; secondly, the conservation hypothesis, accounting for 23 % of all empirical studies, postulates causality from economic growth to energy consumption. The third hypothesis, termed the feedback hypothesis, delineates bidirectional causation and is prevalent in 27 % of studies. Lastly, the neutrality hypothesis, signifying the absence of causality, is present in 21 % of investigations [9].

In the specific context of renewable energy utilization and economic growth, 40 % of empirical examinations supported each of the neutrality, conservation, and growth hypotheses. This underlines the complexity and variability in the relationship between renewable energy consumption and economic growth, warranting further nuanced analysis to discern the intricate interplay of factors influencing this association [10].

Table 1 is illustrated the studies about the link between renewable energy and other independence variables as follows.

**Table 1:** Summary of the empirical results.

No	Authors	Period	Methods	Sample	Findings
1	Bui Minh, T. and H. Bui Van [9]	1995–2009	Vector Error-Correction Model (VCM) and Granger causality	Vietnam	Renewable energy and economics growth (+)
2	Balsalobre and Lorente [8,11,12]	1985–2016	Panel least squares	Germany, France, Italy, Spain, UK	Support for N-shaped EKC REC and CO2 emissions (−)
3	Chen et al. [[13], [14], [15]]		ARDL, FMOLS, DOLS	China	Support for EKC hypothesis, REC and CO2 emissions (−)
4	Wang et al. [[16], [17], [18], [19], [20]]	1980–2011	Vector Error-Correction Model (VCM) and Granger causality	170 countries	Varying results based on income per capita levels of countries.
5	Yang et al. [21]	1995–2014	VCM and panel-ARDL	24 countries in Silk Road Economic Belt (SREB)	Support for EKC REC and CO2 emissions (−) NREC and CO2 emissions (+)

In the context of renewable energy consumption, FDI has been shown to play a crucial role in facilitating the adoption and diffusion of clean energy technologies, particularly in developing countries with limited domestic resources and capabilities [8]. FDI can also contribute to renewable energy consumption by providing financial resources, technical expertise, and market access for renewable energy projects and ventures [4,5,8,11,12]. Fadly studied the green industry in Vietnam [22]. He focuses the important of decreasing the environmental pollution by using the standards of environment management of SMEs. The green industry can help down the CO_2_ emission. Fernandes et al. referred the Urban Metabolism-Based Approaches for Promoting Circular Economy in Buildings Refurbishment [23]. They noted that the circular economy could help reduce the environmental pollution. The circular economy promotes to use more the renewable energy in reality.

Industries are a major source of direct CO2 emissions through the combustion of fossil fuels for energy production and the release of CO2 from industrial processes. This issue includes emissions from manufacturing, power generation, and transportation related to the industry. The type of industry, its energy sources, and its energy efficiency can greatly affect the amount of CO2 it emits [24,25].

Firth et al. researched the Dynamics of Soil Organic Carbon and CO_2_ Flux under Cover Crop and No-Till Management in Soybean Cropping Systems of the Mid-South, United State of America [26]. These research noted that the Crop in Mid-South USA crease more the CO_2_ emission. Flammini et al. concluded the green production to reduce the CO_2_ emission [27].

Hu et al. referred the Effects of Cover Crops and Soil Amendments on Soil CO_2_ Flux in a Mississippi Corn Cropping System on Upland Soil. Their research noted that the CO_2_ emission up by cover crops and soil amendments [28]. Huang et al. studied the Renewable Energy and CO_2_ Emissions: Empirical Evidence from Major Energy-Consuming Countries. They concluded that the renewable energy could reduce the environmental pollution [29]. The more the renewable energy each country use, the less the CO_2_ emission.

Johnathon et al. studied a Proposed Hedge-Based Energy Market Model to Manage Renewable Intermittency. They give some solutions to manage efficiency the renewable energy market [30]. Joo et al. referred the Interaction between FDI, Host Country Characteristics and Economic Growth? A New Panel Evidence from Brics. They noted that the relationship of FDI inflows and economic growth in Brazil, Russia, India and China [31]. They concluded that the FDI inflows and economic growth can make the environmental pollution [31].

Khan et al. focus on the Innovations, Energy Consumption and CO_2_ Emissions in the Global World Countries: An Empirical Investigation [32]. The study show the relationship between energy consumption, innovations and CO_2_ emission in the global economy.

Le et al. referred the Foreign Direct Investment, Environmental Pollution and Economic Growth—an Insight from Non-Linear Ardl Co-Integration Approach. They conclude that the FDI and environmental pollution can promote the economic growth [33]. Liem et al. studied the Reduction in Greenhouse Gas Emission from Seedless Lime Cultivation Using Organic Fertilizer in a Province in Vietnam Mekong Delta Region [34]. They note that the use of organic fertilizer can reduce the greenhouse gas emission and down the environmental pollution.

Madani et al. discuss about Patterns of Emergency Room Visits for Respiratory Diseases in New York State in Relation to Air Pollution, Poverty and Smoking [35]. They noted about the relationship between the environmental pollution and poverty and smoking.

Martí-Ballester et al. have the questions about Do Renewable Energy Mutual Funds Advance Towards Clean Energy-Related Sustainable Development Goals? They concluded that the renewable energy mutual funds could help to invest more in renewable energy and promote to develop the green economy and reduce the CO_2_ emission [36].

Nguyen studied about Carbon Emissions Versus Value-Added in Export-Driven Countries: Case of Vietnam. He noted the more the export of goods and services in Vietnam, the less the CO_2_ emission [37].

Nguyen Thi Ngoc studied about Regional Determinants of FDI Location in Vietnam. She concluded that the FDI inflows can up the environmental pollution in Vietnam [38].

Nguyen et al. referred Financial Development and Renewables in Southeast Asian Countries—the Role of Organic Waste Materials. They concluded that the important role of organic waste material and renewable energy in Southeast Asian Countries to reduce the environment pollution and CO_2_ emission [39].

Overland et al. have the questions are Renewable Energy Sources More Evenly Distributed than Fossil Fuels? They concluded that renewable energy could crease the sustainability development [40].

The Sustainable Development Goals (SDGs) are a set of 17 global goals adopted by the United Nations General Assembly in 2015 as part of the 2030 Agenda for Sustainable Development. The SDGs aim to end poverty, protect the planet, and ensure that all people enjoy peace and prosperity by 2030.

The 17 SDGs are as follows: No Poverty, Zero Hunger, Good Health and Well-being, Quality Education, Gender Equality, Clean Water and Sanitation, Affordable and Clean Energy, Decent Work and Economic Growth, Industry, Innovation and Infrastructure, Reduced Inequalities, Sustainable Cities and Communities, Responsible Consumption and Production, Climate Action, Life Below Water, Life On Land, Peace, Justice and Strong Institutions, Partnerships for the Goals [40]; These goals are interconnected and address a range of economic, social, and environmental issues facing our world today [41]. Achieving these goals requires action and collaboration from governments, businesses, civil society, and individuals around the world [9].

Raihan et al. referred the Nexus between Energy Use, Industrialization, Forest Area, and CO_2_ Emissions: New Insights from Russia. They concluded that the green energy and forest area could reduce the CO_2_ emission in Russia [42].

Raihan et al. also studied the Nexus between Economic Growth, Renewable Energy Use, Agricultural Land Expansion, and Carbon Emissions: New Insights from Peru. They noted that the economic growth could up the CO_2_ emission [43].

Ram et al. studied Energy Transition in Megacities Towards 100 % Renewable Energy: A Case for Delhi. They referred the sustainable development goals toward 100 % green energy in Delhi, India [44].

Shahzad et al. focus on the Export Product Diversification and CO_2_ Emissions: Contextual Evidences from Developing and Developed Economies. They concluded that the export product could up the CO_2_ emission in some countries [45].

Shang et al. studied the Impact of Climate Policy Uncertainty on Renewable and Non-Renewable Energy Demand in the United States. They noted that the US government should focus the policies to greater use the renewable energy consumption [46].

Stjepanovic et al. referred a New Database on Green GDP; 1970–2019: A Framework for Assessing the Green Economy. They concluded that the green economy could be promoted by using renewable energy consumption [47].

Sun et al. studied about the sustainable development in China. They concluded that the greater the use of renewable energy consumption, the better the environment protection [48].

Thu et al. studied the Factors Affecting Environmental Pollution for Sustainable Development Goals—Evidence from Asian Countries. They focus on the renewable energy can promote the sustainable and green economy in Asia [49,50].

Tsai et al. studied the impacts of Environmental Certificate and Pollution Abatement Equipment on SMEs’ Performance: An Empirical Case in Vietnam [51].

Usman et al. referred the Effects of Domestic Material Consumption, Renewable Energy, and Financial Development on Environmental Sustainability in the Eu-28: Evidence from a Gmm Panel-Var [52].

Visconti et al. studied Spontaneous Plants Improve the Inter-Row Soil Fertility in a Citrus Orchard but Nitrogen Lacks to Boost Organic Carbon [53].

Wang et al. noted that Green Finance and Technological Innovation in Heavily Polluting Enterprises: Evidence from China [16]. Wang et al. also studied Spatial Matching Analysis and Development Strategies of County Night-Time Economy: A Case of Anning County, Yunnan Province [18].

Wu et al. referred the Carbon-Neutral Energy Consumption and Emission Volatility: The Causality Analysis of Asean Region. They concluded that the renewable energy consumption can reduce the environmental pollution in ASEAN countries [54].

You Wei et al. studied Types of Environmental Public Interest Litigation in China and Exploration of New Frontiers [55]. Zhang et al. referred the Research on the Coordinated Development of Economic Development and Ecological Environment of Nine Provinces (Regions) in the Yellow River Basin [21].

In summary, the recent research does not focus on using Cobb- Douglas model to estimate the factor affecting the renewable energy consumption at Asian 06 developed countries. Therefore, the paper focuses on the Using Cobb-Douglas Model to analyses Factors affecting the renewable energy consumption – The case of Asian Countries. The data and research methodology is presented in the following section.

### 2.2 Research gap analysis and contributions

Research gap in renewable energy consumption such as while numerous studies have investigated the technical aspects of renewable energy adoption, there is a noticeable gap in understanding the socioeconomic factors influencing the uptake of renewable energy technologies. Further research is needed to explore how factors such as income levels, education, and cultural values affect the acceptance and integration of renewable energy sources within different communities and regions.

Despite the implementation of various renewable energy policies and incentives, there is a dearth of comprehensive assessments regarding their effectiveness. Research in this area should focus on evaluating the impact of different policy measures on renewable energy consumption, considering both short-term and long-term outcomes. This would contribute to the development of more targeted and efficient policy frameworks.

The integration of renewable energy sources into existing energy grids presents a complex challenge that requires further investigation. Current research has touched on technical aspects, but there is a need for more in-depth studies addressing the economic, regulatory, and infrastructural challenges associated with the seamless integration of renewable energy into conventional power systems.

As technology continues to advance, there is a notable gap in research that explores emerging technologies and innovations in the renewable energy sector. Investigating cutting-edge technologies, such as advanced energy storage systems, smart grids, and next-generation solar panels, can provide insights into their feasibility, scalability, and potential contribution to increased renewable energy consumption.

Renewable energy consumption is a multifaceted issue that requires a holistic understanding encompassing various disciplines. Current research often falls within the confines of specific disciplines, neglecting the benefits of cross-disciplinary collaboration. Future studies should encourage interdisciplinary approaches that merge insights from fields such as engineering, economics, sociology, and environmental science to provide comprehensive solutions.

Understanding and influencing consumer behavior is crucial for the widespread adoption of renewable energy. However, research in this area is often limited, with few studies delving into the psychological, social, and economic factors that shape consumer attitudes and choices regarding renewable energy technologies.

## 3 Data and research methodology

### 3.1 Data

To investigate the nexus of electricity consumption, fossil fuel consumption, FDI inflows, economic growth, and renewable energy consumption in Asian countries, this study employs a panel data analysis approach using data for a sample of Asian countries over a specified period. The data for these variables are sourced from various international organizations and databases, such as the World Bank's World Development Indicators, the United Nations Conference on Trade and Development (UNCTAD), and the International Renewable Energy Agency (IRENA). This paper use the data from World Bank at the period 2000–2020 such as follows:

The renewable energy consumption measured by the total green energy consumed 01 year at each country. It contains the renewable energy such as water, wind, solar energy consumption.

The electricity energy consumption measured by the total electricity consumed 01 year at each country. It contains all the source of the energy such as coal, water, wind, fossil fuel and solar energy consumption. The unit of it is TWh.

The fossil fuel energy consumption measured by the total fossil fuel energy consumed 01 year at each country. It contains the un-green energy such as fossil fuel, coal electricity.

FDI inflows are measured by the foreign direct investment inflows to each country at the end of the year. The unit of it is US dollars.

The GDP (Gross Domestic Product) measured the economic growth. The unit of it is US dollars.

### 3.2 Research methodology

The methodological procedures employed in this investigation encompass a sequential set of crucial steps, systematically executed in accordance with the natural logarithm growth rate. These procedural steps are delineated for elucidation purposes. In the context of this inquiry, a Panel Vector Autoregressive (PVAR) model, characterized by its capacity to treat all variables as endogenous, is deployed to mitigate fixed effects in the cross-country manifestations of renewable energy, economic growth, and associated variables within Asian nations. The PVAR model, well-established in the scholarly discourse for scrutinizing policy transmissions and interrelations among diverse economic variables, presents a commendable amalgamation of attributes intrinsic to both standard Vector Autoregressive (VAR) models and Panel data modeling techniques.

The utility of the PVAR model is underscored by its efficacy in addressing endogeneity challenges, characteristic of the VAR family, and concurrently enhancing the precision of estimation by leveraging the panel structure. Furthermore, the application of Panel VAR facilitates an examination of interactions among variables of interest through impulse response functions. Notably, it enhances the efficiency of panel Granger causality analysis within the PVAR framework, thereby aiding in the discernment of the causal linkages between the variables under investigation.

#### 3.2.1 Econometric model and variables

The main variables included in the analysis are electricity consumption (measured in kilowatt-hours per capita), fossil fuel consumption (measured in metric tons of oil equivalent per capita), FDI inflows (measured as a percentage of GDP), economic growth (measured by GDP growth rate), and renewable energy consumption (measured by the share of renewables in total energy consumption).

#### 3.2.2 Estimation techniques

The panel data analysis allows for the estimation of the dynamic relationships among the variables of interest, taking into accounts both cross-sectional and time-series dimensions. The study employs various panel data estimation techniques, such as fixed-effects and random-effects models, as well as panel cointegration tests to examine the long-run relationships among the variables.

#### 3.2.3 Correlation analysis

The correlation analysis reveals the pairwise relationships among the variables and provides preliminary evidence of the interrelationships among electricity consumption, fossil fuel consumption, FDI inflows, economic growth, and renewable energy consumption in Asian countries.

#### 3.2.4 Panel data regression analysis

The panel data regression analysis results reveal significant relationships among the variables of interest, providing insights into the nexus of electricity consumption, fossil fuel consumption, FDI inflows, economic growth, and renewable energy consumption in Asian countries.

**Fig. 1:**
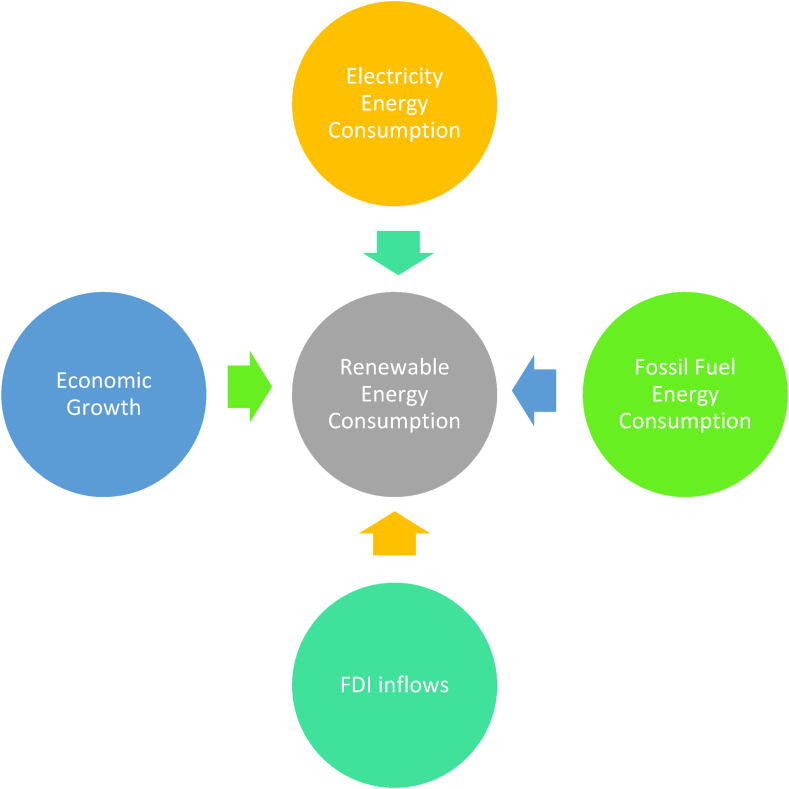
The research diagram. (Source: compiled by the authors)

The research figure is presented at Fig. 1 as follows.

The paper has the hypothesis in the research as follows.

H1 The electricity energy consumption positively affects to the renewable energy consumption.

H2 The fossil energy consumption negatively affects to the renewable energy consumption.

H3 The FDI inflows negatively affect to the renewable energy consumption.

H4 The economic growth negatively affect to the renewable energy consumption.

The independent variable is presented in Table 2 as follows.

**Table 2:** The independent variables using in the Cobb-Douglas regression Model.

Variable	Concept	Relationship
β1	The electricity energy consumption	+
β2	The fossil fuel energy consumption	–
β3	FDI inflows	+
β4	Economics growth	+

## 4 Empirical results

### 4.1 Descriptive statistics

The descriptive statistics provide an overview of the data and highlight the trends and variations in electricity consumption, fossil fuel consumption, FDI inflows, economic growth, and renewable energy consumption across the sample of Asian countries.

The relationship between the FDI inflows and economic growth has many researches in the recently period. However, there is the shortage of the paper referred the nexus between electricity energy consumption, fossil fuel energy consumption, FDI inflows, economic growth and renewable energy consumption. Therefore, the paper chooses this topic and research focus on the 06 Asian developed countries. These countries are Japan, Korea, Singapore, Hong Kong, Israel and China. The study aims to analysis the factors effecting the environment in 06 Asian developed countries. The describe statistic is presented in Table 3 as follows.

**Table 3:** The describe statistic of the variable in the Cobb-Douglas regression Model.

Variable	Obs	Mean	Std. dev.	Min	Max
Renewable energy consumption	126	2.87444	3.651905	0	14.24287
Ln electricity energy consumption	126	8.785816	0.4818639	6.97764	9.347778
Ln fossil fuel energy consumption	126	10.6936	0.6558968	9.058302	12.06804
Ln FDI inflows	124	28.59407	1.300247	24.72506	31.00151
Ln GDP	126	27.43318	1.48904	25.22079	30.32041
Ln CO2	126	5.681412	1.945432	3.410223	9.274993

Table 2 show that the data from 2000 to 2020 contains 126 observations. The dependent variable Q means the renewable energy consumption value. The mean of renewable consumption value is 2.87 % compared to the total electricity energy consumption and the maximum value of 14.24 % in 2020 and minimum value of 0 % in 2000.

The independent variable β1 means the electricity energy consumption with the Ln electricity energy consumption value. The mean of Ln electricity energy consumption is 8.785 TWh and the maximum value of 9.35 TWh in 2020 and minimum value of 6.97 TWh in 2000.

The independent variable β2 means the fossil fuel energy consumption with the Ln fossil fuel energy consumption value. The mean of Ln fossil fuel consumption value is 10.69 million tons and the maximum value of 12.07 TWh in 2020 and minimum value of 9.06 TWh in 2000.

The independent variable β3 means the FDI inflows with the Ln FDI inflows value. The mean of Ln FDI inflows is 28.59 billion US dollars and the maximum value of 31 billion US dollars in 2020 and minimum value of 24.49 billion US dollars in 2000.

The independent variable β4 means the GDP with the Ln GDP value. The mean of Ln GDP is 27.43 billion US dollars and the maximum value of 30.3 billion US dollars in 2020 and minimum value of 25.22 billion US dollars in 2000.

**Fig. 2:**
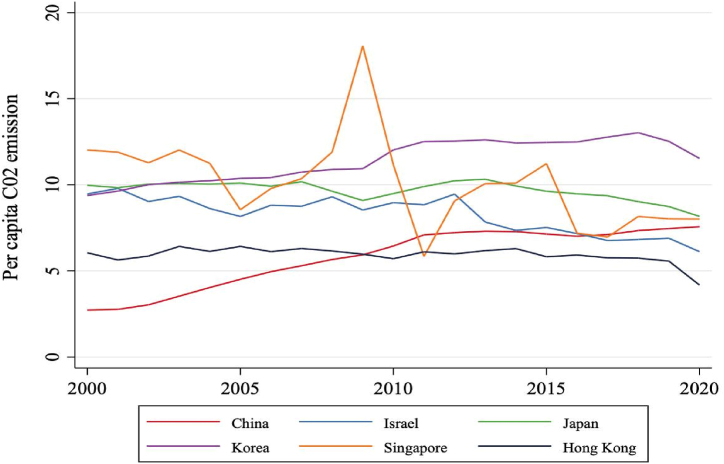
The CO_2_ emission per capita for 06 Asia developed countries from 2000 to 2022 (unit: tons). (Sources: complied by author).

The CO_2_ emission per capita in 06 Asian developed countries is presented in Fig. 2. In general, the CO_2_ emission per capita is stable for 20 year and it reduced in the recent years due to the policy makers announced the sustainable development goals of the United Nation. Everybody in society cares about to protect the environment in these countries.

The CO_2_ emission per capita in Singapore up greater in 2008–2010, after that it reduce in the next years and the CO_2_ emission of Singapore down in recent year.

The CO_2_ emission per capita in Hong Kong is lowest value compared to 05 other countries and it also down in 2018 to present. The CO_2_ emission per capita in Korea is highest value compared to Hong Kong, China, Singapore, Japan and Israel. However, it tends to reduce in recent year.

The CO_2_ emission per capita in China tends to up a little bit for 20 years. However, it still smaller value than Singapore, Israel and Korea.

**Fig. 3:**
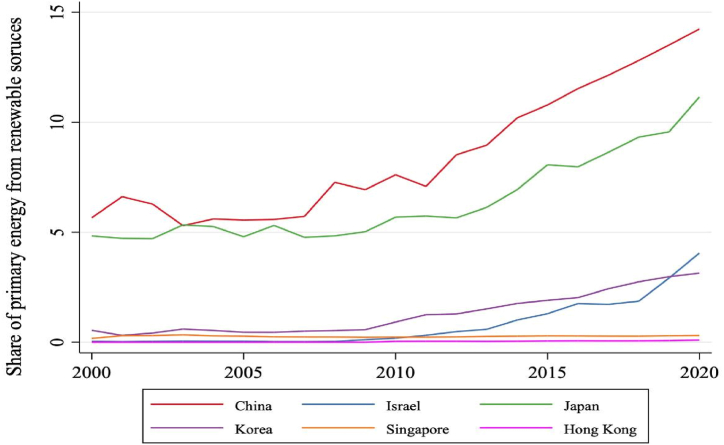
The share of renewable energy consumption compared to the electricity energy consumption for 06 Asia developed countries from 2000 to 2022 (unit: tons). (Sources: complied by author).

In general, all 06 Asian developed countries have the CO2emission per capita tend to down in the recent times. Fig. 3 illustrated the percentage of renewable energy consumption compared to the electricity energy consumption for the period 2000 to 2020 in 06 Asian developed countries. In general, the percentage of renewable energy consumption tend to up for 20 years. China has the percentage of renewable energy consumption at highest value of 15 % in 2020 and Korea has the percentage of renewable energy consumption at lowest value of 3 %.

Table 4 displays the correlation coefficients for the independent variables within the model. The observed correlation coefficients between these variables are minimal, and the variance inflation factor (VIF) remains below 5 for all variables. This low VIF signifies an absence of multicollinearity within the model.

**Table 4:** The correlation of the independence variables in the model.

	Electricity	Fossil Fuel	FDI inflows	GDP
Electricity	1			
Fossil Fuel	0.18	1		
FDI inflows	0.12	0.15	1	
GDP	0.15	0.16	0.12	1

The research results of the dependence (Q) and independence variables are presented in Table 5. The study uses the random effects and Cobb- Douglas model to resolve the research questions.

**Table 5:** The Cobb-Douglas regression model between renewable energy consumption (Q) and the electricity energy consumption, fossil fuel energy consumption, FDI inflows, GDP for 06 Asian developed countries from 2000 to 2020.

Random-effects GLS regression Number of obs = 124						
Group variable: id Number of groups = 6						
R-sq: Obs per group:						
within = 0.3190 min = 19						
between = 0.9638 avg = 20.7						
overall = 0.8458 max = 21						
Wald chi2(7) = 636.40						
corr(u_i, X) = 0 (assumed) Prob > chi2 = 0.0000						
Renewable Energy	Coef.	Std. Err.	z	P > z	[95 % Conf.	Interval]
Lnelectricity	0.598578***	0.922074	0.65	0.0005	2.40581	1.208655
LnFossil Fuel	−0.25578 **	0.504614	−0.51	0.0020	−0.7332465	1.244806
LnFdi Inflows	0.821774***	0.190224	4.32	0.0000	0.448943	1.194606
LnGdp	2.726557***	0.542909	5.02	0.0000	1.662474	3.79064
_cons	−55.7156***	6.392731	−8.72	0.0000	−68.2451	−43.18605

Note: ***P < 0.01, **P < 0.05, *P < 0.1.

The R-squared overall equal 0.8458 shows that 84.58 % the change of independent variables can explain the change of dependence variable.

The P-value equal 0.0005 show that there is the relationship between the electricity energy consumption and renewable energy consumption in 06 Asian developed countries.

The P-value equal 0.020 show that there is the relationship between the fossil fuel energy consumption and renewable energy consumption in 06 Asian developed countries.

The P-value equal 0.0000 show that there is the relationship between the FDI inflows and renewable energy consumption in 06 Asian developed countries.

The P-value equal 0.0000 show that there is the relationship between economic growth and renewable energy consumption in 06 Asian developed countries.

The research results of dependence variable Q-renewable energy consumption and β1- The electricity energy consumption, β2- The fossil fuel energy consumption, β3- The FDI inflows, β4-economic growth in equation (08) as follows:

(1) $$\mathrm{Ln}\left(Q_{i,t}\right)=-55.7156+0.60\;\mathrm{Ln}\left(\beta_{1i,t}\right)\;-0.26\;\mathrm{Ln}\left(\beta_{2i,t}\right)+0.82\;\mathrm{Ln}\left(\beta_{3i,t}\right)+2.72\;\mathrm{Ln}\left(\beta_{4i,t}\right)+e_{i,t}$$

The paper computes the elasticity of renewable energy consumption to the electricity energy consumption E = 0.60. The research results show that if the electricity energy consumption ups 1 % then the renewable energy consumption would up 0.60 %. These results prove that 06 Asian developed countries such as China, Hong Kong, Singapore, Korea, Japan and Israel care about the green electricity.

The paper computes the elasticity of CO_2_ emission to the fossil fuel energy consumption E = 0.924. The research results show that if the fossil fuel energy consumption ups 1 % then the CO_2_ emission would up 0.9 %. These results also prove that 06 Asian developed countries such as China, Hong Kong, Singapore, Korea, Japan and Israel consume almost the environmental polluted fossil fuel electricity. It creases greater CO_2_ emission.

The paper computes the elasticity of renewable energy consumption to the fossil fuel consumption E = −0.26. The research results show that if the fossil fuel energy consumption decreases 1 % then the renewable energy consumption would up 0.26 %. These results prove that 06 Asian developed countries such as China, Hong Kong, Singapore, Korea, Japan and Israel care about the using the green energy. Policy makers and citizens consume greater the green energy to reduce the fossil fuel energy consumption.

The paper computes the elasticity of renewable energy consumption to the FDI inflows E = 0.82. The research results show that if the FDI inflows up 1 % then the renewable energy consumption would up 0.82 %. These results prove that 06 Asian developed countries such as China, Hong Kong, Singapore, Korea, Japan and Israel almost attracted the green FDI inflows that protect the environment. The green FDI inflows promote the greater the renewable energy consumption.

The paper computes the elasticity of renewable energy consumption to economic growth E = 2.72. The research results show that if economic growth ups 1 % then the renewable energy consumption would up 2.72 %. These results prove that 06 Asian developed countries such as China, Hong Kong, Singapore, Korea, Japan and Israel focus almost the green and sustainability development. These economies creases the greater renewable energy consumption compared to the other economies. The greater the economic growth is, the better the green environment is. This is the excellent experiences for the other countries to up the economy and protect the environment.

## 5 Computational experiment and case study

While the benefits are substantial, implementation may pose challenges. Initial setup costs, integration into existing infrastructure, and intermittency issues associated with some renewable sources are factors that require careful consideration. Engineering teams need to conduct thorough feasibility studies to ensure seamless integration.

Company Huanghe Hydropower Hainan Solar Park, a manufacturing firm, sought to enhance sustainability and reduce energy costs. The engineering team implemented a model of renewable energy consumption with a focus on solar power.

The engineering team conducted a comprehensive analysis of the facility's energy needs and assessed the feasibility of integrating solar panels.

Based on the analysis, they opted for solar panels due to the site's ample sunlight. Advanced solar technologies were chosen for optimal efficiency.

The team developed a phased integration plan to minimize disruptions. This included the installation of solar panels, requisite electrical infrastructure upgrades, and implementation of smart grid technologies for efficient energy distribution.

A 30 % reduction in annual energy costs within the first year. Substantial decrease in carbon emissions, aligning with corporate sustainability goals. Positive stakeholder response, enhancing the company's image in the industry.

Ongoing monitoring and optimization are crucial for maximizing benefits. Collaborative efforts between engineering, finance, and management are essential for successful implementation. The model of renewable energy consumption, exemplified by Company Huanghe Hydropower Hainan Solar Park, demonstrates that while challenges exist, the benefits in terms of cost savings, environmental impact, and enhanced corporate image make the effort worthwhile. With meticulous planning and a commitment to sustainability, engineering teams can effectively implement renewable energy solutions in practical settings, contributing to a greener and economically viable future.

This research has the conclusions and recommendations in the below section.

## 6 Conclusion and policy recommendation

### 6.1 Findings

The paper concludes by summarizing the main findings and their implications for energy policy and sustainable development in Asian countries. The study offers recommendations for Asian countries to address the interconnected issues of electricity consumption, fossil fuel consumption, FDI inflows, economic growth, and renewable energy consumption and transition towards a more sustainable energy future.

Electricity energy consumption and economic growth: as countries develop and become more industrialized, they typically require more energy to power their economies. This can lead to higher electricity consumption, particularly in energy-intensive sectors such as manufacturing and transportation. However, this relationship is not always linear, as some countries may prioritize energy efficiency and renewable energy development to reduce their dependence on fossil fuels and mitigate the impacts of climate change.

Fossil fuel energy consumption and CO_2_ emissions: burning fossil fuels such as coal, oil, and natural gas releases CO_2_ into the atmosphere, which contributes to global warming and climate change. Countries that rely heavily on fossil fuels for energy are therefore likely to have higher greenhouse gas emissions than those that prioritize renewable energy and energy efficiency measures.

Renewable energy consumption and greenhouse gas emissions: renewable energy sources such as solar, wind, and hydropower generate electricity without releasing greenhouse gases. Countries that invest in renewable energy development are therefore likely to have lower greenhouse gas emissions than those that rely heavily on fossil fuels. The research results show that the 06 Asian developed countries have the successful using the renewable energy consumption to protect the environment and down the CO_2_ emission.

FDI inflows and greenhouse gas emissions: foreign direct investment can bring capital, technology, and expertise to a country, but it can lead to up energy consumption and greenhouse gas emissions if the investment is directed towards industries that rely heavily on fossil fuels. The research results prove that the 06 Asian developed countries attracted the green and sustainability FDI flows. Therefore, the greater the FDI inflows in these countries are, the better the environment is.

The relationships between electricity energy consumption, fossil fuel energy consumption, FDI inflows, economic growth, and renewable energy consumption are interconnected and complex. These relationships can influence each other in various ways, and understanding these linkages is essential for formulating effective energy and economic policies. Here is an overview of the key relationships:

Electricity energy consumption and economic growth:

A positive relationship exists between electricity consumption and economic growth. As an economy grows, the demand for electricity increases to support industrialization, urbanization, and the expansion of commercial and residential sectors. Conversely, reliable and affordable electricity supply can contribute to higher productivity levels, which can promote economic growth.

Fossil fuel energy consumption and economic growth:

Similar to electricity consumption, fossil fuel consumption is also positively related to economic growth. As countries develop and industrialize, they tend to consume more fossil fuels to meet their energy needs. However, excessive reliance on fossil fuels can lead to negative externalities, such as environmental pollution and greenhouse gas emissions, which can have adverse effects on long-term economic growth and sustainable development.

FDI inflows and economic growth:

Foreign direct investment (FDI) inflows can positively influence economic growth by providing capital, technology, and expertise to the host country. FDI can help improve productivity levels, facilitate technology transfer, and create employment opportunities, which can contribute to economic growth.

FDI inflows and renewable energy consumption:

FDI inflows can play a crucial role in promoting renewable energy consumption by providing financial resources, technical know-how, and market access for renewable energy projects and ventures. FDI can help facilitate the adoption and diffusion of clean energy technologies, particularly in developing countries with limited domestic resources and capabilities.

Renewable energy consumption and economic growth:

The relationship between renewable energy consumption and economic growth can be both positive and negative. On one hand, increasing the share of renewables in the energy mix can contribute to energy security; reduce dependence on fossil fuels, and lower greenhouse gas emissions, which can have positive long-term effects on economic growth. On the other hand, in the short term, the costs of implementing renewable energy technologies can be high, and the transition to a low-carbon economy might have some negative impacts on certain sectors or regions.

Electricity energy consumption, fossil fuel energy consumption, and renewable energy consumption:

These three variables are interrelated in complex ways. As countries aim to reduce their reliance on fossil fuels and increase renewable energy consumption, the dynamics of electricity consumption will also change. The transition to renewable energy sources can lead to changes in the electricity generation mix and influence the overall energy consumption patterns.

In summary, the relationships between electricity energy consumption, fossil fuel energy consumption, FDI inflows, economic growth, and renewable energy consumption are interconnected and can influence each other in various ways. Understanding these relationships can help policymakers formulate effective strategies to promote sustainable energy consumption and economic growth.

The relationships between electricity energy consumption, fossil fuel energy consumption, FDI inflows, economic growth and renewable energy consumption are complex and interconnected. Countries that prioritize sustainable development and invest in renewable energy, energy efficiency, and low-carbon technologies are likely to see long-term economic and environmental benefits.

### 6.2 Implication and application of the study model

Applicability of the Model of Renewable Energy Consumption in Engineering Settings Real-Life Engineering Application:

The model of renewable energy consumption holds substantial applicability in real-life engineering settings, particularly for companies seeking sustainable energy solutions. One pertinent example lies in the optimization of energy consumption within manufacturing facilities. By employing this model, engineers can analyze the feasibility and benefits of integrating renewable energy sources, such as solar panels or wind turbines, into their energy portfolio.

Implementing renewable energy sources can lead to significant cost savings over time. Solar panels, for instance, can generate electricity and reduce a company's reliance on conventional energy sources, mitigating the impact of fluctuating energy prices.

Companies adopting renewable energy models contribute to environmental sustainability by reducing carbon footprints. This aligns with increasing societal and regulatory pressures for businesses to operate in an eco-friendly manner.

Embracing renewable energy solutions positively influences a company's reputation. Consumers are increasingly favoring environmentally responsible businesses, and a commitment to renewable energy can enhance a company's brand image.

### 6.3 Policy recommendations

The policy recommendations for promoting renewable energy consumption involve a comprehensive and strategic approach. The empirical results showed the key recommendations for policy makers as follows:

Implement financial incentives such as tax credits, subsidies, and grants to encourage individuals, businesses, and industries to invest in renewable energy technologies. Establish feed-in tariffs or power purchase agreements that guarantee a fixed payment rate for renewable energy producers, fostering a favorable investment climate.

Develop and enforce clear and consistent regulations that facilitate the integration of renewable energy into the existing energy grid. Establish renewable energy standards or quotas to mandate a certain percentage of total energy production to come from renewable sources.

Allocate resources for research and development in renewable energy technologies to drive innovation and reduce costs. Support partnerships between research institutions, private sector entities, and government agencies to accelerate the development and deployment of new technologies.

Invest in the development and modernization of renewable energy infrastructure, including smart grids, energy storage systems, and efficient transmission networks. Facilitate the integration of decentralized renewable energy systems, such as rooftop solar panels and small-scale wind turbines.

Develop training programs to enhance the skills of the workforce in the renewable energy sector.

Foster education and awareness campaigns to inform the public, businesses, and industries about the benefits and opportunities associated with renewable energy.

Engage in international collaborations to share best practices, technological advancements, and policy experiences related to renewable energy. Participate in global initiatives and agreements aimed at addressing climate change and promoting sustainable energy practices.

Invest in research and infrastructure for energy storage technologies to address intermittency issues associated with certain renewable sources, such as solar and wind. Develop policies that encourage the deployment of energy storage systems at both centralized and decentralized levels. Foster community engagement in renewable energy projects through participatory approaches, such as community-owned solar or wind farms. Encourage local governments to adopt policies that support community-based renewable energy initiatives.

Implement green procurement policies that prioritize the purchase of energy-efficient and renewable energy technologies by government agencies and public institutions. Set an example by leading in the adoption of renewable energy in public buildings and facilities.

Develop long-term energy plans that outline clear targets for increasing the share of renewable energy in the overall energy mix. Regularly review and update policies to reflect advancements in technology and changes in the energy landscape.

These recommendations, when integrated into a comprehensive policy framework, can contribute to the sustainable growth of renewable energy consumption and advance the transition to a cleaner and more resilient energy system.

## 7 Limitations and future recommendations

Future recommendations for promoting and enhancing renewable energy consumption should align with emerging trends, technological advancements, and the evolving needs of the energy landscape.

Invest in research and development to drive continuous innovation in renewable energy technologies, with a focus on improving efficiency, reducing costs, and overcoming existing limitations. Prioritize the development of advanced energy storage solutions to address the intermittency of renewable sources, thereby ensuring a stable and reliable energy supply.

Embrace digital technologies and implement smart grids to optimize the integration of renewable energy into the existing energy infrastructure, enabling better management of supply and demand. Encourage the growth of decentralized energy systems, such as microgrids and community-based renewable projects, to enhance energy resilience and empower local communities.

Explore and promote hybrid energy systems that combine multiple renewable sources, along with energy storage and backup options, to create more robust and versatile energy solutions. Accelerate the electrification of sectors such as transportation and heating, leveraging renewable energy sources to reduce reliance on fossil fuels and decrease overall carbon emissions.

Foster international collaboration on renewable energy projects and explore opportunities for cross-border energy trade to create a more interconnected and resilient global energy system. Integrate circular economy principles into renewable energy practices, emphasizing the recycling and responsible disposal of materials used in renewable technologies.

Develop policies that are flexible and adaptable to evolving technological and market dynamics, ensuring that regulatory frameworks remain conducive to innovation and growth. Consider the implementation of carbon pricing mechanisms and other market-based incentives to further encourage the transition to renewable energy and discourage carbon-intensive practices.

Facilitate public-private partnerships to leverage the strengths of both sectors, fostering collaboration in research, development, and the deployment of renewable energy projects. Continue and expand educational initiatives to raise awareness about the benefits of renewable energy, building public support and encouraging informed decision-making.

Integrate resilience planning into renewable energy strategies, considering potential climate impacts and designing systems that can withstand and recover from disruptions. Ensure that renewable energy policies address social equity considerations, aiming to minimize disparities in access to and benefits from renewable energy resources. Establish robust monitoring and evaluation frameworks to assess the effectiveness of renewable energy policies and make data-driven adjustments as needed.

These future-oriented recommendations underscore the importance of adaptability, collaboration, and ongoing innovation in shaping a sustainable and resilient renewable energy future.

This paper has some limitations such as the research do not refer the relationship between the forest areas and innovation to renewable energy consumption as Khan et al.; Raihan et al. The authors would like to research these issues at 06 developed countries in the future.

## Institutional review board statement

Not applicable.

## Informed consent statement

Not applicable.

## Data availability statement

Not applicable.

## CRediT authorship contribution statement

**Pham Xuan Hoa:** Funding acquisition, Formal analysis, Data curation. **Vu Ngoc Xuan:** Writing – review & editing, Writing – original draft, Visualization, Validation, Supervision, Software, Resources, Project administration, Methodology, Investigation, Funding acquisition, Formal analysis, Data curation, Conceptualization. **Nguyen Thi Phuong Thu:** Writing – original draft, Validation, Supervision, Software, Resources.

## Declaration of competing interest

We wish to confirm that there are no known conflicts of interest associated with this publication.

We confirm that the paper has not been published previously, that is also not under consideration for publication elsewhere.

We confirm that we have given due consideration to the protection of intellectual property associated with this work and that there are no impediments to publication, including the timing of publication, with respect to intellectual property. In so doing we confirm that we have

followed the regulations of our institutions concerning intellectual property.

We further confirm that any aspect of the work covered in this manuscript that has involved either experimental animals or human patients has been conducted with the ethical approval of all relevant bodies and that such approvals are acknowledged within the manuscript.

All procedures followed were in accordance with the ethical standards of the responsible committee on human experimentation (institutional and national) and with the Helsinki Declaration of 1975, as revised in 2000 (5). Informed consent was obtained from all participants for being included in the study.

## Appendix

The research uses the function as equation (1) following:

(2) Q=f(β1,β2,β3,β4…)

In which, Q is the renewable energy consumption-the dependence variable; it measured the using of the wind, solar and water electricity; the unit of it is TWh and percentage of the electricity energy consumption.

β1 is the electricity energy consumption-the independence variable; the unit of it is TWh.

β2 is the fossil fuel energy consumption-the independence variable; the unit of it is TWh.

β3 is FDI inflows-the independence variable; the unit of it is USD dollars

β4 is the Gross Domestic Product (GDP)- the independence variable; the unit of it is USD dollars.

The paper uses the Cobb-Douglas model to estimate the CO_2_ emission as equation (2) following:

(3) $$Q_{i,t}=A\text{.}{\beta 1}_{i,t}^{\alpha 1}{\beta 2}_{i,t}^{\alpha 2}{\beta 3}_{i,t}^{\alpha 3}{\beta 4}_{i,t}^{\alpha 4}+\varepsilon_{i,t}$$

In which, the research could estimate the elasticity of the renewable energy consumption to the independent variable (β1). This variable is the electricity energy consumption as equation (3) as follows:

(4) $$E_{\beta 1}=\frac{dQ_{i,t}}{d{\beta 1}_{i,t}}x\;\frac{{\beta 1}_{i,t}}{Q_{i,t}}=A.\alpha 1.\;{\beta 1}_{i,t}^{\alpha 1-1}{\beta 2}_{i,t}^{\alpha 2}{\beta 3}_{i,t}^{\alpha 3}{\beta 4}_{i,t}^{\alpha 4}.\frac{\beta 1}{A.{\beta 1}_{i,t}^{\alpha 1}{\beta 2}_{i,t}^{\alpha 2}{\beta 3}_{i,t}^{\alpha 3}{\beta 4}_{i,t}^{\alpha 4}}=\alpha 1$$

In which, the research could estimate the elasticity of the renewable energy consumption to the independent variable (β2). This variable is the fossil energy consumption as equation (4) as follows:

(5) $$E_{\beta 2}=\frac{dQ_{i,t}}{d{\beta 2}_{i,t}}x\;\frac{{\beta 2}_{i,t}}{Q_{i,t}}=A.\alpha 2.\;{\beta 1}_{i,t}^{\alpha 1}{\beta 2}_{i,t}^{\alpha 2-1}{\beta 3}_{i,t}^{\alpha 3}{\beta 4}_{i,t}^{\alpha 4}\frac{\beta 2}{A.{\beta 1}_{i,t}^{\alpha 1}{\beta 2}_{i,t}^{\alpha 2}{\beta 3}_{i,t}^{\alpha 3}{\beta 4}_{i,t}^{\alpha 4}}=\alpha 2\;$$

In which, the research could estimate the elasticity of the renewable energy consumption to the independent variable (β3). This variable is the FDI inflows as equation (5) as follows:

(6) $$E_{\beta 3}=\frac{dQ_{i,t}}{d{\beta 3}_{i,t}}x\;\frac{{\beta 3}_{i,t}}{Q_{i,t}}=A.\alpha 3.\;{\beta 1}_{i,t}^{\alpha 1}{\beta 2}_{i,t}^{\alpha 2}{\beta 3}_{i,t}^{\alpha 3-1}{\beta 4}_{i,t}^{\alpha 4}\frac{\beta 3}{A.{\beta 1}_{i,t}^{\alpha 1}{\beta 2}_{i,t}^{\alpha 2}{\beta 3}_{i,t}^{\alpha 3}{\beta 4}_{i,t}^{\alpha 4}}=\alpha 3$$

In which, the research could estimate the elasticity of the renewable energy consumption to the independent variable (β4). This variable is the economic growth as equation (6) as follows:

(7) $$E_{\beta 4}=\frac{dQ_{i,t}}{d{\beta 4}_{i,t}}x\;\frac{{\beta 4}_{i,t}}{Q_{i,t}}=A.\alpha 4.\;{\beta 1}_{i,t}^{\alpha 1}{\beta 2}_{i,t}^{\alpha 2}{\beta 3}_{i,t}^{\alpha 3}{\beta 4}_{i,t}^{\alpha 4-1}.\frac{\beta 4}{A.{\beta 1}_{i,t}^{\alpha 1}{\beta 2}_{i,t}^{\alpha 2}{\beta 3}_{i,t}^{\alpha 3}{\beta 4}_{i,t}^{\alpha 4}}=\alpha 4$$

In equation (2), the research logarithm both side of the equation, it has equation (07) as following:

(8) $$\mathrm{Ln}\left(Q_{i,t}\right)=A+\alpha_{1}\;\mathrm{Ln}\left(\beta_{1i,t}\right)+\alpha_{2}\;\mathrm{Ln}\left(\beta_{2i,t}\right)+\alpha_{3}\;\mathrm{Ln}\left(\beta_{3i,t}\right)+\alpha_{4}\;\mathrm{Ln}\left(\beta_{4i,t}\right)+e_{i,t}$$

This paper assumes that the above equation illustrated the relationship between dependent variable-the renewable energy consumption (Q) and independent variables. It could be describe as follows:

Q is the renewable energy consumption-the independence variable; the unit of it is TWh and percentage of the electricity energy consumption.

A is the constant. It means that CO2 emission when β1 = β2 = β3 = β4 = 0

β1 is the electricity energy consumption-the independence variable; the unit of it is TWh.

β2 is the fossil energy consumption-the independence variable; the unit of it is TWh.

β3 is FDI inflows-the independence variable; the unit of it is USD dollars.

β4 is the Gross Domestic Product (GDP)- the independence variable; the unit of it is USD dollars.
